# Explaining Experiences, Challenges and Adaptation Strategies in COVID-19 Patients: A Qualitative Study in Iran

**DOI:** 10.3389/fpubh.2021.778026

**Published:** 2022-02-03

**Authors:** Sina Ahmadi, Seyed Fahim Irandoost, Ahmad Ahmadi, Javad Yoosefi Lebni, Mohammad Ali Mohammadi Gharehghani, Nafe Baba Safari

**Affiliations:** ^1^Department of Social Welfare Management, University of Social Welfare and Rehabilitation Sciences, Tehran, Iran; ^2^Social Welfare Management Research Center, University of Social Welfare and Rehabilitation Sciences, Tehran, Iran; ^3^Social Determinants of Health Research Center, Clinical Research Institute, Urmia University of Medical Sciences, Urmia, Iran; ^4^PhD in Educational Technology, Faculty of Psychology and Educational Sciences, Allameh Tabataba'i University, Tehran, Iran; ^5^Health Promotion Research Center, Iran University of Medical Sciences, Tehran, Iran; ^6^PhD in Anthropology, Faculty of Social Sciences, University of Tehran, Tehran, Iran

**Keywords:** COVID-19, patients, experiences, adaptation strategies, qualitative study, Iran

## Abstract

**Objective:**

Getting COVID-19 makes a person confront numerous individual, physical, psychological, family and social challenges. Therefore, the present study was conducted to explain the experiences, challenges and adaptation strategies in patients with COVID-19 in Tehran, Iran.

**Methods:**

The present study was performed with a qualitative approach and phenomenological method among 33 patients with COVID-19. From July 20 to September 21, access to participants and data collection were done in person (15 people) and by phone (18 people) through targeted sampling and snowball and semi-structured interviews. Data management was carried out using MAXQDA-2018 software and its analysis was done by the Colaizzi analysis method. Guba and Lincoln's criteria were also observed to improve the quality of results.

**Results:**

After analyzing the data, two main categories and 17 subcategories were obtained, including (1) experiences and challenges (ignoring the disease, blaming, physical health disorders, mental problems, guilt, and remorse, being blamed, living a life of disappointment and ambiguity, emotional challenges, frustrating reactions from others, helplessness and limitation) and (2) disease adaptation strategies (spirituality, learning about COVID-19, doing valuable and fun activities, participating in treatment, strengthening one's spirit and hope, trying to make up for past mistakes and virtual communication).

**Conclusions:**

Based on the results, interventions and policies such as increasing people's health knowledge and literacy to get acquainted with the symptoms of the disease and prompt referral for diagnosis and treatment, teaching stress and psychological pressure management techniques, instructing families to continue emotional and social support for patients and strengthening and reproducing the strategies patients use, along with teaching disease coping skills, harnessing the potential of cyberspace and the media can make it easier to tolerate illness and get back to life.

## Introduction

COVID-19, which broke out in Wuhan, China, in December 2019, is a new member of a broader family of viruses that originally appeared in 2003, leading to respiratory infections ranging from a simple cold to an epidemic and even a pandemic (1, 2). COVID-19, which is currently spreading worldwide (3, 4), is the world's largest current threat to public health and is expected to be the deadliest epidemic of the last 100 years (5–7). Disease incidence and mortality rates of this virus indicate the far-reaching consequences of the disease in the future. According to official statistics, as of December 24, 2021, the total number of people infected with COVID-19 is more than 279,026,000 and the number of deaths is more than 5,405,000. The United States ranks first with 52,859,000 cases. Iran is one of the countries with a high level of involvement in COVID-19 with 6,181,84 cases and 131,306 deaths (8).

Despite the broad effect of COVID-19 on people's lives, such as poor mental health (9, 10), limited access to systems of social support and services (11, 12), depression and anxiety (13, 14), these consequences are not the same for all people (15). Those suffering from infectious and epidemic disease are among those who suffer the most negative consequences. Infectious and epidemic disease, such as SARS and coronavirus, have wide-range and multifaceted effects on the infected person (16–18).

Cava et al. in a study aimed at examining patients' experiences in the SARS quarantine in Toronto, concluded that feelings of uncertainty about life, feelings of isolation, and coping problems with psychological stress were the most important experiences of patients (16). Lin et al. also studied the experiences of Taiwanese patients in quarantine who were hospitalized due to SARS; the results of their study showed that patients experienced fatigue, discomfort, lack of family support and emotional turmoil after a while (17). In the case of COVID-19, research has shown that anxiety and stress (19, 20), sleep disorders (21, 22), and fear (21, 22) are among the challenges that patients experience. Yao et al. in a study showed that patients with COVID-19 have a low psychological tolerance capacity and, due to the current situation and trend of this disease in the world, are highly prone to psychological disorders such as depression and negative thoughts (23). Shahyad and Mohammadi. also expressed feelings of loneliness, getting labeled, denial, disappointment, and aggression as the experiences of a patient with the new coronavirus (24).

People with COVID-19 face many physical, psychological, and other challenges in life. Therefore, it seems necessary to study their experiences and problems. Most of the studies conducted in this field have been quantitative and experimental; few studies were qualitative and addressed patients' problems from their point of view. While a better qualitative study can penetrate the hidden layers of life experiences, interpretations and perceptions of people with COVID-19. By identifying patients' problems and challenges, they can be better helped to return to life after treatment. Therefore, the aim of this study is to explain the experiences, challenges and adaptation strategies in patients with COVID-19 in Iran with a qualitative approach.

## Methods

### Design

This research was conducted with a qualitative approach and a phenomenological method. This method looks at the world as it is lived by a person and tries to explore the meanings that the person has experienced in daily life and to reach a new understanding of the world of life by revealing new and neglected meanings of these experiences (25).

### Participants

The study population consisted of patients recovered from COVID-19 in Tehran, Iran. Inclusion criteria included experience of getting COVID-19, passing at least 3 weeks after discharge from the hospital or at least 1 month after a positive result of the COVID-19 test, having the right physical condition for the interview, and willingness to participate in the study.

### Data Collection

In order to reach the participants, first targeted sampling was used, and then snowball sampling was used so that after each interview, the researcher asked the participant to introduce other people who met the inclusion criteria to the researcher. After receiving the code of ethics (IR.USWR.REC.1399.231) from the University of Social Welfare and Rehabilitation Sciences, the researchers went to the hospitals (Milad, Lolagar, Imam Khomeini, and Masih Daneshvari) and obtained the telephone numbers and addresses of 13 COVID-19 patients who had previously been hospitalized and recovered. Then researchers contacted them, expressing the objectives and process of the research. Researchers asked them to participate in the research if they would, and after the interview, they were asked to introduce other people. Twenty participants were selected through this process.

Data were collected through semi-structured interviews in person (15 people) and by telephone (18 people). In-person interviews were conducted in the participants' homes and in accordance with health protocols. At the beginning of the interviews, the researcher (First corresponding author of the article) began the interview by introducing himself, giving a brief description of the goals and process of the research, and asking a few demographic questions. The interview question guide was used to continue the interview. The participants were also encouraged to discuss their own experiences with the sickness rather than what they thought knew about the illness, its symptoms, or its characteristics. The interview questions were designed so that the researchers could extract information from the participants' speech and derive it as much as possible from their own experience rather than prior knowledge. The interview Question Guide was approved before the first interview in collaboration with all the authors of the article during several discussion and review sessions (Table 1). However, before the final confirmation, three pilot interviews were conducted by telephone so that the researchers could find out whether the interview questions could extract the information and experiences of the participants properly or not. This refers to whether the interview guide questions are created in accordance with the study objectives and whether the research objectives can be met using these questions. Also, whether they lead to the collection of data on COVID-19 patients' challenges and experiences and the development of relevant strategies. This issue was detected, and the difficulties were remedied after conducting three pilot interviews and analyzing them. All participants were asked the identical questions from the interview question guide, with the exception that the order of the questions was not the same. Following-up and investigation questions were asked based on the participants' responses so that the researchers could extract the hidden layers of the participants' experiences and feelings.

**Table 1:** Interview question guide.

**NO**.	**Questions**
1	How did you find out you had COVID-19? What was your reaction? Explain.
2	How did you feel about yourself and others when you found out you had COVID-19? What were you thinking of?
3	What problems did you face physically and mentally after having COVID-19?
4	How did your family, medical staff and others react and how did you feel about them?
5	What made you more annoyed during the treatment? Explain.
6	What changes have taken place in your relationship with your family members? Explain.
7	How did you calm down after getting COVID-19?
8	During treatment, what strategies did you use to cope with COVID-19? Explain.

The interviews continued until no new codes were found in the data and only the previous codes were repeated. A word or phrase associated with a group or class of objects, events, or behaviors that share some common qualities is referred to as a “code” in qualitative research. One of the steps of analysis in coding is initial coding, in which we analyze the interview text paragraph by paragraph, phrase by phrase, and word by word for anything that could be a concept or term to describe reality as a code. The data gathering procedure was halted when this step was repeated and no new code was retrieved (26). The researchers therefore concluded that the data were saturated and did not continue interviews after the interview No. 33. The time and place of the interviews were determined by the participants, and all interviews were recorded with the consent of the participants. The data collection period lasted from August 20 to September 21, 2020.

### Data Analysis

The second and corresponding authors of the article used MAXQDA-2018 software to organize and analyze data in accordance with the Colaizzi method (27) in this study. The researchers first wrote the interviews after listening to them multiple times, and then went over the text of the interview numerous times, highlighting key phrases and assigning interpretations to them in the form of beginning codes. The researchers then wrote all of the initial codes independently, placing the codes that they considered had a similar meaning in one class and naming the classes. Then, based on the similarity of the concepts, the various classes were amalgamated, and more general classes were developed. The categories and subcategories were finally formed.

### Trustworthiness

In this study, 32 items of Tong qualitative research report were observed (COREQ) (28). Also, Guba and Lincoln criteria (29), which are the most famous quality criteria in qualitative research, were observed in this research. The credibility criterion was obtained by purposefully selecting the participants who had the most differences in terms of demographic characteristics, and researcher's opinion of the interview was checked at the end of the interview. The results of the analysis and coding of the interviews were returned to all participants and their approval was acquired. Since the researchers had experience of conducting several qualitative studies on COVID-19 patients and their families, they could easily communicate with the participants and extract relevant information from them. The viewpoint of qualitative research experts and nurses with expertise caring for COVID-19 patients were employed in coding and data processing to establish the confirmability criterion. Also, all authors' opinions of the article from different scientific disciplines were used at all the stages of the research to obtain the dependability criterion. The categories and subcategories were finalized after consultation with all authors during the analysis and coding process. Again, the Transferability criterion was met via writing the entire research process and providing a comprehensive description of it, using many quotes for each of the subcategories, and giving the results of data analysis and coding to six patients who met the inclusion criteria but did not participate in the study and obtaining their approval.

### Ethical Considerations

In order to observe the ethical considerations, at the beginning of the interviews, the researcher told the participants such things as the goals and necessity of the research, being free to participate in the study, the right to interrupt the interview if they desired and keeping their names secret in publishing the results. Also, during the interview, the researcher observed health issues such as using a mask and gloves, observing the appropriate distance and taking only one face-to-face interview during the day, which was prepared in consultation with specialist doctors.

## Results

Thirty-three people participated in the study, whose demographic information is shown in Table 2. Also, from the data analysis, two categories and 17 subcategories were obtained, which are stated in Table 3.

**Table 2:** Demographic information of participants.

**Variable**	**Group**	**Frequency**
Gender	Femal	15
	Male	18
Age	20–30	8
	31–50	12
	Over 50	13
Education	Under diploma	9
	Diploma to graduate	15
	Higher than graduate	7
Marital status	Marrie	20
	Single	13
Length of hospitalization	<1 week	15
	1–2 weeks	11
	More than 2 weeks	7

**Table 3:** Categories, subcategories and codes obtained from interviews with COVID-19 patients.

**Codes**	**Subcategories**	**Categories**
Experiences and challenges	Ignoring the disease	Denial of the disease, denial of the symptoms of the disease, delay in seeing a doctor, considering the symptoms of the disease as a cold, not observing health issues at the beginning of the disease
	Blaming	Blaming themselves, blaming others, blaming government officials, blaming the Chinese lifestyle
	Physical health disorders	Shortness of breath, high fever, dry and severe cough, delirium, nausea, fatigue and exhaustion, loss of smell and taste, sleep and eating disorders
	Mental health problems	Aggression toward family and medical staff, depression, extreme stress and fear, suicidal ideation, wishing to die, growing negative feelings toward others, tendency to take revenge on society
	Guilt and remorse	Felt guilt and remorse due to not observing health protocols, fell guilty for increasing the burden on nurses and medical staff, feel guilty for disrupting the lives of family members and endangering their health
	Being blamed	Blamed by the medical staff, being blamed by family members, being blamed by friends and relatives
	Living a life of disappointment and ambiguity	Feeling of failure and hopelessness in treatment, prolongation of the treatment process, worry about long-term complications of the disease, ambiguity of burial condition, ambiguity about the future condition of the disease
	Emotional challenges	Disorders in emotional and sexual relationships with spouse, disorders in relationships with children and other family members, disorders in relationships with friends and relatives, distance from family in difficult moments of illness
	Frustrating reactions from others	Not understanding the patient's condition, talking about death and burial before the patient, not having hope for the patient's survival, putting fear and stress into the patient
	Helplessness and limitation	Tired of staying home, tired of prolonged treatment, restrictions on going out, deprivation of daily recreation, restrictions on work activities
disease adaptation strategies	Spirituality	Benediction, prayer, making oblation, donating a part of their property to charity and religious organizations, considering the disease as a test of God and seeing God's wisdom in the creation of the disease
	Increasing knowledge about COVID-19	Learning about ways of COVID-19 transition, learning about COVID-19 treatment, learning about the possible effects of COVID-19, learning about how to care for and quarantine at home
	Doing useful and fun activities	Reading books, doing educational and work projects, watching movies
	Participating in treatment	Caring the other family members, taking medication on time, listening to nurses and doctors, staying in home quarantine, observing health principles, enough eating and sleep
	Strengthening one's spirit and hope	Optimism, avoidance of negative news, avoidance of (ignoring) cyberspace news and rumors, watching hopeful movies, talking with inspiring people, communicating with patients recovered from COVID-19
	Trying to make up for past mistakes	Asking for forgiveness from others, reconciling with others, making up for bad deeds, calling old friends and reviewing memories
	Virtual communication	Making voice and video calls with family members and friends, creating a Telegram group and sharing their experiences

### Experiences and Challenges

The first category obtained from data was the challenges experienced by COVID-19 patients during the disease and the treatment process. These experiences and challenges included ignoring the disease, blaming, physical health disorders, mental health problems, guilt and remorse, being blamed, living a life of disappointment and ambiguity, emotional challenges, frustrating reactions from others, helplessness and limitation.

#### Ignoring the Disease

Many participants said that they initially denied getting the disease and its symptoms and did not want to believe that they had COVID-19, so most of them delayed going to the doctor at the beginning of the disease and did so not follow the principles of quarantine.

“*My illness started with a fever and a cough. My daughters insisted that I go to the doctor. They told me it might be COVID-19, but I did not accept it at all until I fainted from the severity of the illness.” (45-year-old married woman)*.

“*When the doctor told me that I had COVID-19 symptoms and that I most likely got COVID-19, I did not accept his words and even argued with him. I could not believe that I had taken COVID-19 at all.” (30-year-old single man)*.

“*In the beginning, I always thought I had a cold or the flu. I was all fooling myself. I did not want to believe that I got COVID-19 because I was afraid of it.” (28-year-old married woman)*.

In fact, many patients in the early stages of the disease, due to lack of knowledge about the disease and fear of the conditions they have to endure after the diagnosis, try to deny having the disease, which could make treatment conditions much more difficult for them.

#### Blaming

At the beginning of the disease, most participants sought the cause of their disease, some blamed themselves for not observing health issues, and other participants blamed other people, some blamed government officials for lack of proper planning and policy, and ultimately a limited number blamed the Chinese for their lifestyle and diet.

“*Early on, I was always looking for the culprit. I complained a lot to the authorities because I thought it was their fault that I got COVID-19. Maybe if they had quarantined the cities from the beginning, I would not be sick either.”(59-year-old married woman)*.

“*I mostly blame myself for not observing; I traveled.” (62-year-old married man)*.

“*I really observed health protocols, but most people did not comply. It was more their fault. If I were the officials, I would fine anyone who do not comply.” (27-year-old single man)*.

“*I blamed the Chinese for my illness because I thought that their lifestyle and diet made COVID-19 spread around the world.” (62-year-old married woman)*.

In the first stage of the disease, many patients reviewed the circumstances of the last week of their lives before the disease and looked for the ultimate culprit of getting COVID-19, which in most cases they blamed others.

#### Physical Health Disorders

All participants reported that COVID-19 severely affected their health. Although the consequences of the disease were different in the participants, most of them had problems such as shortness of breath, high fever, severe and dry cough, delirium, nausea, extreme fatigue, sleep and eating disorders, and loss of smell and taste.

“*Sometimes I felt suffocated. It was a terrible time. I had many coughs. After I got well, I lost my sense of smell for a long time.” (45-year-old married woman)*.

“*My illness started with a high fever, so much so that I was all delusional, thinking I was dying.” (65-year-old married man)*.

“*My sleep was disturbed; my appetite became very low.” (26-year-old single woman)*.

In many patients, COVID-19 disrupted their health and caused them many physical problems that in some cases lasted for weeks or even months.

#### Mental Health Problems

Living with COVID-19 affects all aspects of patients' lives, so that most of them face psychological problems that may prolong their recovery process. Some participants stated that they could not control themselves during the illness due to physical and psychological pressures and behaving aggressively toward family members and medical staff. Others said they had thought about suicide many times during the treatment process and had wished for death. Also, because some participants blamed others for their illness, it caused them to develop negative feelings so that they tended to seek revenge from society.

“*When I was hospitalized, I was very nervous. I always complained to the nurses and argued with them. When I came home, I got into fights with my family members. They took good care of me, but I shouted at them all. It was not my fault. I was in a very bad mood (68-year-old married woman)*.

“*I was terrified at the beginning, I was very stressed, I was mentally disturbed.” (29-year-old single man)*.

“*When I found out I had COVID-19, I hated everyone. I wanted to kill all the idiots who do not observe health issues. For a while, I thought of going out and infecting many idiots outside. I wanted to take revenge on them.” (26-year-old single woman)*.

Getting COVID-19 imposes a heavy psychological burden on patients due to the conditions and limitations it creates, which can result in many psychological problems for them and further lead to risky behaviors.

#### Feelings of Guilt and Remorse

Most participants felt guilt and remorse due to not observing health protocols, increasing the burden on nurses and medical staff, disrupting family members lives, and endangering their health.

“*I always blame myself for not observing (the health protocols) so as not to take COVID-19. I am very sorry. If I get well, I will observe (the health protocols) very well.” (37-year-old married man)*.

“*I felt guilty to see nurses and doctors being so annoyed and working so hard because of my irresponsibility and people like me.” (52-year-old married man)*.

“*I bothered my family a lot during this time, I disrupted their lives and endangered their health, so I do not feel good about myself, I wish I observed more.” (21-year-old single woman)*.

A person with COVID-19 can put a lot of pressure on the treatment staff and the patient's family, so in some cases, patients blame themselves and feel remorse and guilt.

#### Being Blamed

Most of the participants stated that they were blamed by the medical staff, family members, friends and relatives for having COVID-19 and were faced with the stigma of irresponsibility.

“*Instead of supporting and comforting me, my family always blamed me for not taking care of myself, and that bothered me.” (25-year-old single man)*.

“*The medical staff was exhausted. They worked hard for the patients, but sometimes they blamed us and said why did we not follow the health issues.” (37-year-old married woman)*.

“*Most of my friends and relatives, who called me, bothered me with their words rather than comforted me, repeating that it was my fault because of not observing (the health protocols).” (21-year-old single woman)*.

Since observing health protocols could significantly reduce the risk of getting COVID-19, in cases where people got COVID-19, friends and relatives and even some medical staff would stigmatize them as irresponsible and blame them if they followed health protocols, they were very unlikely to get COVID-19.

#### Living With Disappointment and Ambiguity

Due to the lengthy treatment process and no definitive cure for COVID-19, many participants expressed that they were in despair and saw a vague future ahead of them. Also some were concerned about unknown complications of COVID-19, the conditions after death, and the manner of burial.

“*The nurses always gave me hope; they told me that I would get well, but I was completely disappointed and felt I would never get better again.” (67-year-old married man)*.

“*The first time I was told that I had COVID-19, I thought it would take a week, but I was sick for about 4 weeks. I felt distraught. I ran out of patience.” (38-year-old married woman)*.

“*When I saw that doctors themselves did not know much about this disease, I became more worried. Sometimes I heard that the effects of COVID-19 may last for the rest of my life.” (69-year-old single woman)*.

“*What bothered me more than the illness itself was thinking about the afterlife. Everything was vague. It was not at all clear how we would be buried if we died.” (80-year-old married man)*.

The state of the disease, as well as the conditions that might affect the patient's body after death and prevent them from being buried according to the customs, were distressing for most patients and made their lives frustrating and worrying because they were concerned about their health while also worried about how they would be buried after death.

#### Emotional Challenges

One of the most important problems that COVID-19 patients had and suffered from was the disorder in their emotional relationships with family members, especially spouses and children. Also, most patients suffered from disorders in relationships with friends and relatives, and they were upset that they were alone in the difficult moments of illness when they needed support more than ever.

“*I haven't slept with my wife for almost a month and this bothers me.” (40-year-old married man)*.

“*It bothered me that I could not see my children up close. Even now that I am well, I talk to my children with fear and worry.” (52-year-old married woman)*.

“*It's been a long time since I saw any of my friends and relatives up close. This situation is annoying. I got very lonely.” (26-year-old single man)*.

The consequent conditions of getting COVID-19 and quarantine, forcing the patient to be alone for several weeks and not see anyone up close, became an emotional challenge for many people, leaving them longing for family and friends.

#### Frustrating Reactions From Others

Most of the participants stated that those around them did not understand the patient's condition and gave them more stress and frustration with their words.

“*Many my associates, when they found out I had COVID-19, acted as if I was going to die in a few days, and that bothered me.” (55-year-old married man)*.

“*Even though I was still alive and well, my family thought I was going to die, so they were worried about the funeral all the time, and I heard them talking about it.” (80-year-old married man)*.

“*When I got worse a little, my family was distraught. They would come and ask me how I was; they didn't allow me to rest at all. As soon as my eyes fell asleep, they came and called me and woke me up for fear that I would die.” (70-year-old married woman)*.

Many families did not receive adequate training in how to care for a COVID-19 patient and had a lot of fear and stress in taking care of a COVID-19 patient, and sometimes they transmitted this fear and anxiety to the patients and made their condition worse.

#### Helplessness and Limitations

Prolonged treatment process caused many patients to feel tired and helpless about the situation and also distance themselves from many of their daily activities and recreation, and this issue put much pressure on patients.

“*It's tough for me to stay home all the time and do nothing. When I think about how my work is behind schedule, I get angrier.” (30-year-old single man)*.

“*Being sick for a few weeks and then not being able to go out for a few weeks after it gets better bothers me a lot, I really ran out of patience.” (33-year-old married woman)*.

“*A lot of my work is behind schedule. I do not know how to do it. This disease really disturbed my whole life.” (55-year-old married man)*.

“*Before the disease broke out, I used to go to the mountains and go out, but now I'm all at home, and it bothers me.” (28-year-old single man)*.

The prolongation of the treatment process and the subsequent quarantine, as well as the forced stay at home, had exhausted many participants and made them feel helpless.

### Disease Adaptation Strategies

Many COVID-19 patients reduced the severity of their condition during the illness and the recovery process by relying on specific behaviors and strategies, making them more relaxed and better able to cope with the disease.

#### Spirituality

Some patients, due to their religious beliefs, tried to cope with this disease through doing things such as benediction, prayer, making oblation, donating a part of their property to charity and religious organizations, considering the disease as a test of God and seeing God's wisdom in the creation of the disease.

“*When I found out I had COVID-19, I was terrified, and I was always in benediction to God and I was praying that He would give me another chance so that I could be a more useful person.” (55-year-old married man)*.

“*I prayed at nights that I could survive, and I vowed to give part of my property to the poor and charities if I got rid of the disease.” (70-year-old married woman)*.

“*I was a religious person, so from the first day I found out I had COVID-19, like the rest of my life, I tried to look at it as a test of God, and I was sure God saw something in it that I got sick, so I was ready for everything, even for death.” (40-year-old married man)*.

In fact, having a religious view of the disease and doing religious work has been used by most patients as a strategy to reduce the stress and anxiety caused by COVID-19, and in many cases can positively affect adapting to occurring conditions.

#### Increasing Knowledge About COVID-19

After getting COVID-19, most patients tried to better cope with the situation by increasing their knowledge about transitions, treatment, consequences, and how to take care of themselves at home.

“*When I took COVID-19, I was searching a lot about how it would be transmitted because I was worried about my family's health.” (28-year-old single man)*.

“*I was trying to help myself by raising my knowledge about treatment options, such as boiling and drinking some herbal teas.” (21-year-old single woman)*.

“*When I lost my sense of smell, I was very scared, but I started studying, which I finally realized was normal and would get better over time. Maybe if I had not studied, I would be much more worried about this.” (45-year-old married woman)*.

“*At first I did not know how to take care of myself, but by asking others and also reading various sites, I was able to get good information.” (64-year-old married man)*.

Because at the beginning of the disease, many people had relatively little knowledge about COVID-19, some patients tried to increase their knowledge of the disease by studying and searching various sites, which helped them to cope with it more easily.

#### Doing Valuable and Fun Activities

To cope with the conditions caused by COVID-19 and stay at home, some patients tried to make conditions more tolerable and even more productive by doing useful and entertaining activities such as reading books, doing educational and work projects and watching movies.

“*When I had to stay home, I read books all the time and I was both entertained and had fun.” (33-year-old married woman)*.

“*While I was sick, I tried not to fall behind in my work. I wrote two chapters of my dissertation and did other things.” (25-year-old single man)*.

“*I tried to entertain myself. Sometimes I watched movies and sometimes I did things that didn't need to go out online.” (33-year-old married man)*.

As the treatment process and quarantine for patients became longer, some of them tried to take advantage of the situation and work on their backlogs or entertain themselves so as not to be mentally disturbed.

#### Participation in the Treatment of the Disease

However, COVID-19 imposed a lot of psychological pressure on patients, some patients tried to better participate in the treatment process by controlling their mental condition and following all medical instructions to get better and reducing the burden on family members as much as they could.

“*I tried to do most of my work, I did not let my family members be bothered.” (69-year-old married woman)*.

“*I could cope with the disease very soon and I told myself, this disease is like other diseases, that if I take good care of myself, I will get well-soon, so I listened to the nurses and doctors.” (59-year-old married woman)*.

“*After I got sick, I tried to plan my diet and sleep according to what the doctors said and followed all their advice to get well-soon.” (66-year-old married man)*.

“*I tried very quickly to cope with the disease and think about treatment and improving my health, so I did everything I thought could be good for my health in those circumstances.” (45-year-old married woman)*.

In fact, unlike most participants, some participants were able to control themselves after the disease and participate appropriately in their treatment process by following all the recommendations of doctors and nurses, which could affect the improvement of their health.

#### Strengthening One's Spirit and Hope

Some patients tried to keep their spirits up and fight the disease better by avoiding negative news, not paying attention to cyberspace news and rumors, watching hopeful movies, talking with inspiring people, and communicating with patients recovered from COVID-19.

“*In early days of getting infected, I was very scared because horrible things were being posted on the internet, so I tried to visit cyberspace very little.” (45-year-old married woman)*.

“*I mostly tried to give myself hope by watching hopeful movies and talking with people who made me feel good.” (28-year-old single man)*.

“*When I got sick, I was very worried. A friend of mine introduced me to his brother, who had taken COVID-19 and recovered. He helped me a lot. His words made me hopeful.” (44-year-old married man)*.

“*After getting infected, I tried to pay less attention to negative news, such as the COVID-19 death rate, because every time I heard about the number of people died of COVID-19 in Iran, my mood got worse.” (52-year-old married woman)*.

With the spread of COVID-19 in Iran, so much false news was spread in cyberspace that patients might lose their spirits when they saw this news. Therefore, many patients tried to stay away from cyberspace to strengthen their spirits and have more relationships with people who had spirits. The connection with the survivors of COVID-19 could significantly impact their spirits and hope.

#### Trying to Make Up for Past Mistakes

Some patients said that they saw themselves very close to death when they were sick, so out of fear or anything else, they tried to reduce the psychological burden of illness by doing things like asking for forgiveness from others, reconciling with others, making up for evil deeds, calling old friends and reviewing memories.

“*At first I thought I might die, so I tried to ask all those whom I thought were upset with me to forgive me and get reconciled with those whom I cut off.” (70-year-old married woman)*.

“*When I got sick, I thought a lot about it and felt that I was not a good person, so I tried to be a good person from then on. I called many friends I had been unware of for years and asked how they were.” (45-year-old married man)*.

“*I was a person in my life who got nervous very quickly and people around me were always upset of me. After realizing that I took COVID-19, I came to my senses and tried to change my behavior, become a good person, and win the hearts of those around me.” (28-year-old single man)*.

In fact, COVID-19 makes patients fear death, which is why many patients try to feel good by doing valuable things, compensating for past mistakes, and feeling more comfortable so that they will suffer less in the hereafter if they died.

#### Virtual Communication

Staying at home and away from the outside imposed much psychological pressure on patients, so some tried to cope with the illness more easily via cyberspace. They used cyberspace to communicate and to share their experiences.

“*After being discharged from the hospital, I was alone at home. I was very annoyed. I could not see my children, but we talked using a WhatsApp video call, and this calmed me down a lot.” (33-year-old married woman)*.

“*When I got COVID-19, many relatives were worried about me, so when I got better, I created a Telegram group and added all my friends and relatives, and I always kept in touch with them. They gave me a lot of encouragement.” (Mr. 30-year-old single)*.

“*From the first day I tried to share everything I experience, I saw that others really liked it and always commented on me. In general, I think the cyberspace helped me a lot to be able to endure the hard days of illness.” (40-year-old married man)*.

In fact, having COVID-19 and being forced to stay away from others paved the way for the proper use of cyberspace, and many patients could make quarantine conditions more bearable for themselves with the help of virtual communications which could reduce their loneliness and worry. In fact, people who have been infected with COVID-19 and have been forced to stay away from others have turned to cyberspace to compensate for lost direct communication, and many patients have been able to make quarantine conditions more bearable for themselves through virtual communication, which has had a significant impact on reducing feelings of loneliness and anxiety.

## Discussion

The present study identified the experiences, challenges, and adaptation strategies of COVID-19 patients in Tehran, Iran with a qualitative approach to better deal with this disease and its suffering. The results showed that patient' challenges with the disease's physical and psychosocial dimensions increased its burden. Although some of these challenges are specific to the duration of treatment and their effects are somewhat reduced after recovery, individuals struggle with some of them for a long time.

One of the challenges for COVID-19 patients was ignoring the disease, not observing health issues such as using a mask, and delaying its diagnosis and treatment, making it more prevalent and difficult to prevent its spread. Rong et al. showed that early detection and shortening the waiting time for diagnosis cannot eliminate COVID-19, but can significantly reduce the risk of transmission and more effectively prevent the spread of COVID-19 (30). Various studies indicate that some people do not follow the health tips, such as wearing a mask, to prevent COVID-19, while the use of masks and social distance can significantly reduce the prevalence of the disease (31, 32). In general, accepting the disease and trying to observe the health principles and seeing a doctor can effectively break the chain of the disease and reducing its complications, such as hospitalization and mortality, and this issue depends on the acceptance of the disease by patients.

In the present study, another experience of COVID-19 patients was self-blame or attributing their condition and illness to other people, officials, and China. This issue is expressed not only by COVID-19 patients but also by the general public. People look for the culprit in epidemics, being forced for quarantine, and being away from others. Lack of investment on prevention by health officials and systems around the world is one of the reasons for the prevalence of COVID-19 (33). Given that the origin of the COVID-19 virus is still unknown, speculation continues about the cause of the virus, and some consider eating habits in China to be the medical cause of the virus, leading to pessimism about Chinese people; a negative attitude toward Chinese immigrants has increased since the outbreak of the COVID-19 pandemic (34). In the Barreneche study, the culprits for the current situation and the prevalence of COVID-19 are China and its unhealthy eating and cooking habits, irresponsible people who do not stay home, and people who can pay for travel and bring the virus (35). However, the culprit of the outbreak of COVID-19 can be anyone or any institution or any country, but the fundamental point is the attempt to observe health issues and help prevent them, which should be considered by people infected or at risk.

Patients with COVID-19 had many physical problems. Shortness of breath, high fever, dry and severe cough, delirium, nausea, exhaustion, loss of smell and taste, sleep and eating disorders are some of the problems that these people struggle with and cause disorders in their physical health. These features and problems have been reported in various studies on COVID-19 (22, 36–39). These physical problems will also damage patients' mental health and their relationships with family and associates, and because of their distance from others during the disease quarantine period, they are very harmful and highlight the need for post-recovery care.

The results showed that infected people had various psychological problems such as depression, suicidal ideation, negative feelings toward others, aggression, severe negativity and disappointment, consistent with previous research in this field (9, 40, 41). Mousavi-Almaleky et al. in a study by examining the psychological and spiritual dimensions of patients with COVID-19 showed that disappointment, anger and hatred toward others are among the challenges these people face (42). Some of these psychological problems occur due to blaming others and trying to find the culprit and take revenge for the cause of the patient's condition, and partly due to the physical injuries and deaths of patients similar to the patient. This situation highlights the need for special attention to the mental health of COVID-19 patients and their mental rehabilitation, as the patient may harm others after recovery or may tend to infect others during the disease.

Feelings of guilt and remorse after getting COVID-19 were another challenge for the people in the present study. Given that observing health guidelines essentially prevents getting COVID-19 (43), not observing health guidelines is considered a shortcoming. Failing to comply with protocols and getting COVID-19 increased the burden on the family, nurses, and medical staff, and they were likely to infect others, so they felt guilty and remorseful. In a study by Pellegrini et al. COVID-19 patients felt guilty for not following health instructions and not being in quarantine (44). The children in Idoiaga et al. study also feel guilty for thinking they can infect their grandparents (45). Feelings of guilt can lead to depression and anxiety in the long run and have negative consequences for one's health. This anxiety, stress and worry caused by the illness of themselves and other family members and the possibility of losing them leads to increased levels of stress, depression and slows down the recovery process in these people.

The patients in the present study also experienced the challenge of being blamed by family, medical staff and associates. In the study of Rahmatinejad et al. similar to our results, COVID-19 patients experienced reprimands by others due to not observing protocols and getting infected and expressed the feeling of being pointed and distanced by others as one of their bitter experiences (41). Research by Jakovljevic et al. also showed that victim reprimands increased during COVID-19 (46). This reprimand may, in some cases, lead to the rejection of the patient. Various studies have shown rejection due to COVID-19 (47–49). In general, not understanding the patient's condition, being blamed, and worrying about the judgment of society and even the family may lead to psychological disturbances, social isolation and loneliness of patients.

Another challenge for COVID-19 patients was a life of despair and ambiguity, which included hopelessness in treatment and its complications, the future state of the disease, and ambiguity in the conditions after death. Aliakbari Dehkordi et al. study showed that being desperate for life and the future was one of the challenges for COVID-19 patients (50). In a study examining patients' experiences in SARS quarantine, the results showed that feelings of uncertainty about life and a sense of isolation were the most important experiences of patients (16). Regarding death and subsequent conditions, Rahmatinejad et al. in their study, showed that COVID-19 patients face the stress of death, the condition of burial and not seeing loved ones before death (41). Patients with severe conditions and progressing the disease often have a lower life expectancy and experience a great deal of confusion about death and beyond, and the same is true for COVID-19 patients because death rates cause patients to see themselves at risk of death.

According to the results, another challenge for patients with COVID-19 was emotional challenges, including the disruption of emotional relationships with family members, especially spouses and children, and the reduced emotional support from others. Studies have shown that having a person with COVID-19 puts a lot of stress on family members and disrupts family members' relationships with the person (51, 52). It can be said that because this virus is contagious and one of the ways to prevent its transmission is to observe social distance, infected people cannot receive enough support and empathy, and this, in some cases, slows down the healing process. According to the results of studies, social support makes people resistant to the psychological consequences of COVID-19 and increases their tolerance and endurance (34, 53). The need for emotional support leads family members to have a closer relationship with the patient. However, at the same time, the warnings and concerns about the possibility of transmitting the virus in close contact with the patient make family members and relatives scared and worried, and their behaviors appear contradictory and inappropriate. They turn away from the person in a sensitive situation.

One of the challenges mentioned by COVID-19 patients was facing adverse and disappointing reactions. In the study of Rahmatinejad et al. impatience as one of the negative emotional reactions of family members was one of the unpleasant experiences of patients (41). While the patient needs empathy and encouragement, family members may cause more stress and strain to the patient by not understanding his condition and despairing of his recovery. These conditions threaten the patient's recovery and return to life, and may even prepare the ground for his death.

The study results showed that the helplessness and limitations of staying at home and the inability to do things in life are other challenges that these people face and result in chronic fatigue. In a 2020 study by Rahmatinejad et al. boredom and loneliness generated by prolonged quarantine caused by getting infected made patients bothered (41). In the Iheduru—Anderson study, 2020, nurses also experienced burnout and helplessness due to prolonged illness and difficult working conditions (54). Helplessness refers to situations in which persons becomes disappointed with any improvement in their affairs and any controls over them, and succumbs to frustration and despair. This is also the case for COVID-19 patients, who get tired of the vague and uncertain situation in which they find themselves.

Given the condition of COVID-19 and its long-term consequences, adaptation is a process that takes place over time. This adaptation is associated with changes in a person's lifestyle that require planning. Various studies show that these plans should be tailored to new situations and guide the patient in using appropriate adaptation strategies (55). The present study results showed that since adaptation to this disease is multifaceted and affected by different factors, patients use different strategies to deal with it. One of the strategies used among the patients was spirituality. Various studies have shown that in times of social crisis and natural disasters, a closer relationship with God can facilitate tolerating the condition (56–58). In the study of Danhauer et al. spiritual-religious adaptation was one of the main methods used to create adaptation to the disease (59). This issue is more significant in Iran due to the religious beliefs of the Iranian people, and they, by relying on God, as emphasized in religious teachings, calm down and endure difficult situations better.

Another interesting result of the research was the knowledge-building strategy about COVID-19 and trying to find ways to deal with it from various sources. Gaining knowledge about transmission, treatment, effects, and consequences of the disease and understanding how to care for and quarantine at home are things that people take from various sources such as books and cyberspace. This is significant given that COVID-19 patients have multiple challenges, and it can be used as a model for future and other diseases, because this strategy allows the patient to participate in disease control and prevention.

One exciting result of the study was the performance of valuable and entertaining activities by COVID-19 patients. These activities included reading, watching movies, and doing work and study tasks. In line with the previous strategy, the importance of this approach can also be emphasized, because doing fun and useful activities can be a tool against frustration and fatigue caused by the disease, and while improving patients' mental health, accelerate the process of recovery and return to life, makes the negative aspects of the disease less vital to them.

Participating in the treatment of the disease, including observing the health principles, adequate nutrition and sleep, and staying in home quarantine to take care of other family members, were among the other strategies utilized by the infected people. Consistent with this finding, Mohammadzadeh, in his study, showed that the rate of adherence to medication, diet, physical activity, and preventive issues related to COVID-19 is higher than other diseases (60). Although COVID-19 puts a lot of stress on patients, some try to participate better in the treatment process by controlling their condition and follow all medical instructions to get better. This can be both because of fear and an attempt to compensate for their role in infecting and to assist family, medical staff, and physicians.

Another challenge for COVID-19 patients to cope with was strengthening sprit and hope. In the study by Danhauer et al. a hopeful outlook on the future and creating a distance between oneself and the disease were the main methods used to adapt to the disease (59). One of the ways to strengthen the spirit is not to use or not pay attention to cyberspace and its various news and rumors. Venegas-Vera et al. reported that one of the main shortcomings of social media and cyberspace is the ability to quickly disseminate misinformation that can lead to confusion and distraction (61). In the study of Ahmad and Murad, social media has a significant impact on the spread of fear and panic caused by the outbreak of COVID-19 in Iraqi Kurdistan and has a potentially negative impact on the mental health and well-being of individuals (62). Therefore, this strategy is also essential to deal with the disease and accelerate the person's recovery because despair and loss of spirit cause the person to become physically incapacitated and practically lose the ability to compete with the disease. Ways to strengthen this spirit should be highlighted in the person, and the grounds for losing the spirit should be eliminated.

As mentioned earlier, people blame themselves after getting the disease, and most of the time, they reproach themselves for not following the health tips. In this regard, one of the strategies used by patients was seeking forgiveness, trying to compensate for evil deeds. Given that COVID-19 is a dangerous infectious disease that in many cases leads to death (63), people consider themselves close to death, and therefore the review of the past and the mistakes increases.

One of the strategies patients used to maintain emotional and social support was video calls with family members and friends and sharing their experiences in cyberspace. Researches have shown that the use of internet can help improve the condition of patients by exchanging information (61, 64, 65). The prevalence of COVID-19 has been concurrent with building new Internet and cyberspace experience. Given that social distancing is an important principle in this period, people turn to virtual spaces and social networks to compensate for this forced sedentary life and isolation and maintain their connection with the outside world. The use of cyberspace in different sections of society, especially patients, has dramatically helped implement social distancing and cope with COVID-19 effectively.

## Limitations and Strengths

This study is one of the few studies that qualitatively examines the challenges, experiences and adaptation strategies for COVID-19 from the perspective of patients, which can provide helpful information to policy makers, psychologists, social workers and health activists to identify and understand the steps to reduce patients' problems and provide the conditions for their return to life. The findings of the strategies that patients employed to adapt to the disease and the situations in which they were found to be one of the novel components of the current study. Iranian studies have not considered these strategies, and the present study is distinctive in this field. Another strength of this study was that two of the article's authors had the experience of getting COVID-19 and therefore had a better understanding of the issue and were better able to work on coding and data analysis.

The study also had limitations; this included scheduling the interview, which was one of the study's main limitations because patients had difficulty in determining the time of the interview due to the complications of the disease, and the time of the interview changed with their opinion. This implies that, despite our best efforts, some participants (patients) had to postpone their interviews owing to COVID-19 issues, and we had to wait until the next opportunity to interview them. Another key disadvantage was that data analysis and coding might be prejudiced because two researchers had previously encountered Covid-19; researchers examined and encoded the data in groups to address this constraint. It was done under the observation of all the article's authors to ensure no bias in the data analysis. Another limitation was that due to the prevalence of COVID-19, some participants were reluctant to be interviewed in person. The researcher solved their concerns by observing health issues and using a mask, and in some cases, he used phone interviews. Observing health issues for the interview, such as keeping a safe distance and wearing a mask, in some cases made the voices of the respondents not loud enough during the recording of the interview, and the researchers asked the participants to speak louder. Because the patients and the researcher wore masks and kept an appropriate distance during the interview, the recorded voices were of poor quality in some circumstances, and the researcher could not hear the sound well, forcing the researcher to advise the patients to speak louder. Researchers had to listen to the recorded sound several times to avoid missing a word in some scenarios due to the low quality of the voices. All speeches, however, were assessed and coded in general, and no information was lost. Finally, the participating women tended to have female interviewers to more easily share their experiences with the researcher. Hence, the researchers used a trained female researcher who was familiar with the principles of interviewing and qualitative research.

## Conclusion

The results showed that COVID-19 patients confront multiple and multifaceted challenges such as ignoring the disease, blaming, physical, psychological and emotional problems, feelings of guilt and remorse, being blamed and disappointment and ambiguity. They use the following strategies to cope with these conditions and overcome these challenges: spirituality, increasing knowledge about COVID-19, doing valuable and fun activities, participating in the treatment of illness, strengthening one's spirit and hope, trying to make up for past mistakes, and virtual communication. Based on the results of this study, interventions and policies should be multi-level and diverse. Increasing people's health knowledge and literacy to observe health issues and familiarity with the symptoms of the disease and prompt referral for diagnosis and treatment, teaching methods to deal with stress and psychological pressure, educating families to continue emotional and social support of patients, and providing more access to counseling to develop skills for strengthening spirits and relaxation are part of the actions. Furthermore, reinforcing and replicating the strategies used by patients, teaching them coping skills, utilizing the potential of cyberspace to educate and entertain people, and increasing entertainment programs in the media can make it easier to tolerate illness and return to life.

## Data Availability Statement

The raw data supporting the conclusions of this article will be made available by the authors, without undue reservation.

## Ethics Statement

The study was provided ethical approval by the University of Social Welfare and Rehabilitation Sciences (IR.USWR.REC.1399.231). Written consent was obtained from all participants.

## Author Contributions

JY, SI, and SA contributed to designing the study. AA, NB, and SI collected the data and analyzed it by JY, MM, and SI. The final article was written by SI, JY, and MM. All authors participated and approved the study design and read and approved the final manuscript.

## Conflict of Interest

The authors declare that the research was conducted in the absence of any commercial or financial relationships that could be construed as a potential conflict of interest.

## Publisher's Note

All claims expressed in this article are solely those of the authors and do not necessarily represent those of their affiliated organizations, or those of the publisher, the editors and the reviewers. Any product that may be evaluated in this article, or claim that may be made by its manufacturer, is not guaranteed or endorsed by the publisher.

## References

[1] Bao Y Sun Y Meng S Shi J Lu L. 2019-ncov epidemic: address mental health care to empower society. Lancet. (2020) 395:e37–8. 10.1016/S0140-6736(20)30309-3 32043982 PMC7133594

[2] SoleimanvandiAzar N Irandoost SF Ahmadi S Xosravi T Ranjbar H Mansourian M. Explaining the reasons for not maintaining the health guidelines to prevent covid-19 in high-risk jobs: a qualitative study in iran. BMC public health. (2021) 21:1–15. 10.1186/s12889-021-10889-4 33941149 PMC8090924

[3] Yoosefi Lebni J Abbas J Moradi F Salahshoor MR Chaboksavar F Irandoost SF. How the covid-19 pandemic effected economic, social, political, and cultural factors: a lesson from iran. Int J Soc Psychiatry. (2021) 67:298–300. 10.1177/0020764020939984 32615838 PMC8107447

[4] Yoosefi Lebni J Irandoost SF Xosravi T Ahmadi S Ziapour A Soofizad G. Explaining the problems faced by iranian housewives during the covid-19 quarantine period, and their adaption strategies: a qualitative study. Womens Health. (2021) 17:1–13. 10.1177/17455065211063291 34842000 PMC8640282

[5] Williams SN Armitage CJ Tampe T Dienes K. Public perceptions and experiences of social distancing and social isolation during the covid-19 pandemic: a uk-based focus group study. BMJ Open. (2020) 10:e039334. 10.1136/bmjopen-2020-039334 32690752 PMC7387310

[6] Lebni JY Irandoost SF Mehedi N Sedighi S Ziapour A. The role of celebrities during the covid-19 pandemic in iran: opportunity or threat? Dis Med Public Health Prep. (2021). 10.1017/dmp.2020.498. [Epub ahead of print]. 34565495

[7] Lebni JY Ziapour A Mehedi N Irandoost SF. The role of clerics in confronting the covid-19 crisis in iran. J Relig Health. (2021) 60:2387–94. 10.1007/s10943-021-01295-6 34052942 PMC8164828

[8] Worldometers.info. (2021). Available online at: https://www.Worldometers.Info/coronavirus/ (accessed December 24, 2021).

[9] Nobles J Martin F Dawson S Moran P Savovic J. The Potential Impact of Covid-19 on Mental Health Outcomes and the Implications for Service Solutions. Bristol: National Institute for Health Research, University of Bristol (2020).

[10] Li S Wang Y Xue J Zhao N Zhu T. The impact of covid-19 epidemic declaration on psychological consequences: a study on active weibo users. Int J Environ Res Public Health. (2020) 17:2032. 10.3390/ijerph17062032 32204411 PMC7143846

[11] Zahran S Shelley TOC Peek L Brody SD. Natural disasters and social order: modeling crime outcomes in florida. Int J Mass Emerg Dis. (2009) 27:26–52. Available online at: http://ijmed.org/articles/119/

[12] Parkinson D. Investigating the increase in domestic violence post disaster: an Australian case study. J Int Viol. (2019) 34:2333–62. 10.1177/0886260517696876 29294681

[13] Filgueiras A Stults-Kolehmainen M. The relationship between behavioural and psychosocial factors among brazilians in quarantine due to covid-19. SSRN. (2020). 10.2139/ssrn.3566245. [Epub ahead of print].

[14] Finlay JM Kler JS O'shea BQ Eastman MR Vinson YR Kobayashi LC. Coping during the covid-19 pandemic: a qualitative study of older adults across the united states. Front Public Health. (2021) 9:643807. 10.3389/fpubh.2021.643807 33898379 PMC8058195

[15] Mantovani A Dalbeni A Beatrice G. Coronavirus disease 2019 (covid-19): we don't leave women alone. Int J Public Health. (2020) 65:235–6. 10.1007/s00038-020-01369-4 32277247 PMC7147358

[16] Cava MA Fay KE Beanlands HJ McCay EA Wignall R. The experience of quarantine for individuals affected by sars in toronto. Public Health Nurs. (2005) 22:398–406. 10.1111/j.0737-1209.2005.220504.x 16229732

[17] Lin ECL Peng YC Tsai JCH. Lessons learned from the anti-sars quarantine experience in a hospital-based fever screening station in taiwan. Am J Infect Control. (2010) 38:302–7. 10.1016/j.ajic.2009.09.008 20083327 PMC7115330

[18] Willgoss TG Yohannes AM Goldbart J Fatoye F. Everything was spiraling out of control: experiences of anxiety in people with chronic obstructive pulmonary disease. Heart Lung. (2012) 41:562–71. 10.1016/j.hrtlng.2012.07.003 22939112

[19] Bajema KL Oster AM McGovern OL Lindstrom S Stenger MR Anderson TC. Persons evaluated for 2019 novel coronavirus—united states, january 2020. Morb Mortality Weekly Rep. (2020) 69:166. 10.15585/mmwr.mm6906e1 32053579 PMC7017962

[20] To KK-W Tsang OT-Y Yip CC-Y Chan K-H Wu T-C Chan JM-C. Consistent detection of 2019 novel coronavirus in saliva. Clin Infect Dis. (2020) 71:841–3. 10.1093/cid/ciaa149 32047895 PMC7108139

[21] Yang L Wu D Hou Y Wang X Dai N Wang G. Analysis of psychological state and clinical psychological intervention model of patients with covid-19. MedRxiv [Preprint]. (2020). 10.1101/2020.03.22.2004089933796496

[22] Liu S Yang L Zhang C Xiang Y-T Liu Z Hu S. Online mental health services in china during the covid-19 outbreak. Lancet Psychiatry. (2020) 7:e17–8. 10.1016/S2215-0366(20)30077-8 32085841 PMC7129099

[23] Yao H Chen J-H Xu Y-F. Patients with mental health disorders in the covid-19 epidemic. Lancet Psychiatry. (2020) 7:e21. 10.1016/S2215-0366(20)30090-0 32199510 PMC7269717

[24] Shahyad S Mohammadi MT. Psychological impacts of covid-19 outbreak on mental health status of society individuals: a narrative review. J Military Med. (2020) 22:184–92. 10.30491/JMM.22.2.184

[25] Alase A. The interpretative phenomenological analysis (ipa): a guide to a good qualitative research approach. Int J Educ Lit Stud. (2017) 5:9–19. 10.7575/aiac.ijels.v.5n.2p.9

[26] Flick U. (2018). Managing Quality in Qualitative Research. 2nd Edn. SAGE Publications Ltd. 10.4135/9781529716641

[27] Shosha GA. Employment of colaizzi's strategy in descriptive phenomenology: a reflection of a researcher. Eur Sci J. (2012) 8:31–43. 10.19044/esj.2012.v8n27p%p

[28] Tong A Sainsbury P Craig J. Consolidated criteria for reporting qualitative research (coreq): a 32-item checklist for interviews and focus groups. Int J Qual Health Care. (2007) 19:349–57. 10.1093/intqhc/mzm042 17872937

[29] Lincoln YS Lynham SA Guba EG. Paradigmatic controversies, contradictions, and emerging confluences, revisited. Sage Handjournal Qualit Res. (2011) 4:97–128.

[30] Rong X Yang L Chu H Fan M. Effect of delay in diagnosis on transmission of covid-19. Math Biosci Eng. (2020) 17:2725–40. 10.3934/mbe.2020149 32233563

[31] Eikenberry SE Mancuso M Iboi E Phan T Eikenberry K Kuang Y. To mask or not to mask: modeling the potential for face mask use by the general public to curtail the covid-19 pandemic. Infect Dis Model. (2020) 5:293–308. 10.1016/j.idm.2020.04.001 32355904 PMC7186508

[32] Howard MC. Understanding face mask use to prevent coronavirus and other illnesses: development of a multidimensional face mask perceptions scale. Brit J Health Psychol. (2020) 25:912–24. 10.1111/bjhp.12453 32588949 PMC7361913

[33] Rudan I. This is who to blame for the covid-19 pandemic. J Global Health. (2021) 11:03035. 10.7189/jogh.11.03035 33692888 PMC7914402

[34] Xu C Liu Y. Does mislabeling covid-19 elicit the perception of threat and reduce blame? J Behav Public Administ. (2021) 4:1–30. 10.30636/jbpa.42.225

[35] Barreneche SM. Somebody to blame: on the construction of the other in the context of the covid-19 outbreak. Soc Reg. (2020) 4:19–32. 10.14746/sr.2020.4.2.02

[36] Yang Y Shang W Rao X. Facing the covid-19 outbreak: what should we know and what could we do? J Med Virol. (2020) 92:536–7. 10.1002/jmv.25720 32091134 PMC7228352

[37] Struyf T Deeks JJ Dinnes J Takwoingi Y Davenport C Leeflang MM. Signs and symptoms to determine if a patient presenting in primary care or hospital outpatient settings has covid-19. Cochrane Database Syst Rev. (2021) 2: CD013665. 10.1002/14651858.CD013665 33620086 PMC8407425

[38] Arkin N Krishnan K Chang MG Bittner EA. Nutrition in critically ill patients with covid-19: challenges and special considerations. Clin Nutr. (2020) 39:2327. 10.1016/j.clnu.2020.05.007 32425291 PMC7227546

[39] Sallam M Dababseh D Yaseen A Al-Haidar A Taim D Eid H. Covid-19 misinformation: mere harmless delusions or much more? A knowledge and attitude cross-sectional study among the general public residing in jordan. PLoS ONE. (2020) 15:e0243264. 10.1371/journal.pone.0243264 33270783 PMC7714217

[40] Wu Y-T Zhang C Liu H Duan C-C Li C Fan J-X. Perinatal depression of women along with 2019 novel coronavirus breakout in china. Am J Obstet Gynecol. (2020) 223:240.e1–240.e9. 10.2139/ssrn.3539359 32437665 PMC7211756

[41] Rahmatinejad P Yazdi M Khosravi Z Shahisadrabadi F. Lived experience of patients with coronavirus (covid-19): a phenomenological study. J Res Psycholo Health. (2020) 14:71–86. 10.52547/rph.14.1.71

[42] Mousavi-Almaleky Z Ghomian S Baqernejad M. The psychological and spiritual dimensions of covid-19 patients: a qualitative study. Quart Clin Psychol Stud Allameh Tabataba Univ. (2021) 10:1–26. 10.22054/JCPS.2021.55243.243434895184

[43] Wang C Horby PW Hayden FG Gao GF. A novel coronavirus outbreak of global health concern. lancet. (2020) 395:470–3. 10.1016/S0140-6736(20)30185-9 31986257 PMC7135038

[44] Pellegrini V Giacomantonio M De Cristofaro V Salvati M Brasini M Carlo E. Is covid-19 a natural event? Covid-19 pandemic and conspiracy beliefs. Person Ind Differen. (2021) 181:111011. 10.1016/j.paid.2021.111011 PMC9756891 36540625

[45] Idoiaga N Berasategi N Eiguren A Picaza M. Exploring children's social and emotional representations of the covid-19 pandemic. Front Psychol. (2020) 11:1952. 10.3389/fpsyg.2020.01952 32922334 PMC7456920

[46] Jakovljevic M Jakovljevic I Bjedov S Mustac F. Psychiatry for better world: Covid-19 and blame games people play from public and global mental health perspective. Psych Danubina. (2020) 32:221–8. 10.24869/psyd.2020.221 32796790

[47] Kahambing JGS Edilo SR. Stigma, exclusion, and mental health during covid19: 2 cases from the philippines. Asian J Psychiatry. (2020) 54:102292. 10.1016/j.ajp.2020.102292 32682301 PMC7352109

[48] Sotgiu G Dobler CC. Social stigma in the time of coronavirus disease 2019. In: Eur Respiratory Soc. (2020) 56:2002461. 10.1183/13993003.02461-2020 32631833 PMC7338401

[49] Grover S Singh P Sahoo S Mehra A. Stigma related to covid-19 infection: are the health care workers stigmatizing their own colleagues? Asian J Psychiatry. (2020) 53:102381. 10.1016/j.ajp.2020.102381 32882670 PMC7451190

[50] Aliakbari Dehkordi M Eisazadeh F Aghajanbigloo S. Psychological consequences of patients with coronavirus (covid-19): a qualitative study. Iran J Health Psychol. (2020) 2:9–20. 10.30473/IJOHP.2020.52395.1074

[51] Robert R Kentish-Barnes N Boyer A Laurent A Azoulay E Reignier J. Ethical dilemmas due to the covid-19 pandemic. Ann Int Care. (2020) 10:1–9. 10.1186/s13613-020-00702-7 32556826 PMC7298921

[52] Wallace CL Wladkowski SP Gibson A White P. Grief during the covid-19 pandemic: considerations for palliative care providers. J Pain Symptom Manag. (2020) 60:e70–6. 10.1016/j.jpainsymman.2020.04.012 32298748 PMC7153515

[53] Grey I Arora T Thomas J Saneh A Tohme P Abi-Habib R. The role of perceived social support on depression and sleep during the covid-19 pandemic. Psychiatry Res. (2020) 293:113452. 10.1016/j.psychres.2020.113452 32977047 PMC7500407

[54] Iheduru-Anderson K. Reflections on the lived experience of working with limited personal protective equipment during the covid-19 crisis. Nurs Inquiry. (2021) 28:e12382. 10.1111/nin.12382 33010197 PMC7646033

[55] Rehman U Shahnawaz MG Khan NH Kharshiing KD Khursheed M Gupta K. Depression, anxiety and stress among indians in times of covid-19 lockdown. Commun Mental Health J. (2021) 57:42–8. 10.1007/s10597-020-00664-x 32577997 PMC7309680

[56] Glass K Flory K Hankin BL Kloos B Turecki G. Are coping strategies, social support, and hope associated with psychological distress among hurricane katrina survivors? J Soc Clin Psychol. (2009) 28:779–95. 10.1521/jscp.2009.28.6.779

[57] Henderson TL Roberto KA Kamo Y. Older adults' responses to hurricane katrina: daily hassles and coping strategies. J Appl Gerontol. (2010) 29:48–69. 10.1177/0733464809334287

[58] Lebni JY Khorami F Azar FEF Khosravi B Safari H Ziapour A. Experiences of rural women with damages resulting from an earthquake in Iran: a qualitative study. BMC Public Health. (2020) 20:1–13. 10.1186/s12889-020-08752-z 32375725 PMC7201637

[59] Danhauer SC Crawford SL Farmer DF Avis NE. A longitudinal investigation of coping strategies and quality of life among younger women with breast cancer. J Behav Med. (2009) 32:371–9. 10.1007/s10865-009-9211-x 19308716

[60] Mohammadzadeh A. Effectiveness of electronic health care and drug monitoring program to prevent covid-19 and adherence to therapeutic regimen in patients with ischemic heart disease-a pilot study. J Military Med. (2020) 22:139–46.

[61] Venegas-Vera AV Colbert GB Lerma EV. Positive and negative impact of social media in the covid-19 era. Rev Cardiov Med. (2020) 21:561–4. 10.31083/j.rcm.2020.04.195 33388000

[62] Ahmad AR Murad HR. The impact of social media on panic during the covid-19 pandemic in iraqi kurdistan: online questionnaire study. J Med Int Res. (2020) 22:e19556. 10.2196/19556 32369026 PMC7238863

[63] Karmakar M Lantz PM Tipirneni R. Association of social and demographic factors with covid-19 incidence and death rates in the us. JAMA Network Open. (2021) 4:e2036462. 10.1001/jamanetworkopen.2020.36462 33512520 PMC7846939

[64] Cuello-Garcia C Pérez-Gaxiola G van Amelsvoort L. Social media can have an impact on how we manage and investigate the covid-19 pandemic. J Clin Epidemiol. (2020) 127:198. 10.1016/j.jclinepi.2020.06.028 32603686 PMC7320665

[65] Sharif N Opu RR Alzahrani KJ Ahmed SN Islam S Mim SS. The positive impact of social media on health behavior towards the covid-19 pandemic in bangladesh: a web-based cross-sectional study. Diabetes Metab Syndrome Clin Res Rev. (2021) 15:102206. 10.1016/j.dsx.2021.102206 34298272

